# Comparative Transcriptome Analysis Reveals That Lactose Acts as an Inducer and Provides Proper Carbon Sources for Enhancing Exopolysaccharide Yield in the Deep-Sea Bacterium *Zunongwangia profunda* SM-A87

**DOI:** 10.1371/journal.pone.0115998

**Published:** 2015-02-13

**Authors:** Qi-Long Qin, Yi Li, Mei-Ling Sun, Jin-Cheng Rong, Sheng-Bo Liu, Xiu-Lan Chen, Hai-Nan Su, Bai-Cheng Zhou, Bin-Bin Xie, Yu-Zhong Zhang, Xi-Ying Zhang

**Affiliations:** 1 State Key Laboratory of Microbial Technology, Shandong University, Jinan 250100, China; 2 Marine Biotechnology Research Center, Shandong University, Jinan 250100, China; LSU Health Sciences Center School of Dentistry, UNITED STATES

## Abstract

Many marine bacteria secrete exopolysaccharides (EPSs) that have important ecological and physiological functions. Numerous nutritional and environmental factors influence bacterial EPS production. However, the regulatory mechanisms of EPS production are poorly understood. The deep-sea *Bacteroidetes* bacterium *Zunongwangia profunda* SM-A87 can produce high quantities of EPS, and its EPS production is enhanced significantly by lactose. Here, we studied the reasons behind the significant advantage that lactose has over other carbon sources in EPS production in SM-A87. RNA-seq technologies were used to study lactose-regulated genes in SM-A87. The expression level of genes within the EPS gene cluster was up-regulated when lactose was added. Supplement of lactose also influenced the expression of genes located outside the EPS gene cluster that are also involved in EPS biosynthesis. The major glycosyl components of SM-A87 EPS are mannose, glucose and galactose. Genomic metabolic pathway analyses showed that the EPS precursor GDP-mannose can be synthesized from glucose, while the precursor UDP-glucose must be synthesized from galactose. Lactose can provide glucose and galactose simultaneously and prevent glucose inhibition. Lactose can also greatly stimulate the growth of SM-A87. Taken together, lactose acts not only as an inducer but also as a carbohydrate source for EPS production. This research broadens our knowledge of the regulation of EPS production in marine bacteria.

## Introduction

Many marine bacteria secrete high molecular weight exopolysaccharides (EPSs). EPS surrounds bacterial cells and enhances their survival [1,2,3,4,5]. EPS plays many important ecological and physiological roles for bacteria. EPSs can help bacteria to adhere to a surface and form biofilms that provide protection against antibiotics, predation and other challenges [1,6]. Because of its negative charge and propensity to flocculate, EPSs can adsorb and concentrate dissolved organic molecules and trace metals that can be utilized by EPS-secreting bacteria. EPSs can also participate in the formation of marine snow, which transports fixed carbon and nitrogen from the surface to deep waters [1,7]. It has been reported that the EPS secreted by a deep-sea psychrotolerant bacterium can protect bacterial extracellular proteases from autolysis and prevent their diffusion, which may benefit the bacterium [8]. EPSs may act as cryoprotectants for sea-ice bacteria. In one studied case, the EPS secreted by an arctic sea-ice bacterial strain could enhance the salt tolerance of the strain and improve the viability of the strain after several freeze-thaw cycles [5].

Microbial EPS production is affected by many nutritional and environmental conditions, e.g., carbon and nitrogen sources, the carbon/nitrogen ratio, oxygen and aeration rate, and incubation temperature and pH [2,9]. However, the regulatory mechanisms of these factors are rarely studied. *Zunongwangia profunda* SM-A87 (hereafter called SM-A87), a bacterium isolated from deep-sea sediment in the southern Okinawa Trough, yields high quantities of EPS with interesting rheological properties, such as high viscosity and tolerance to high temperatures and salinities [10,11]. This EPS also has potential biotechnological applications in wastewater treatment and oil recovery [12,13]. Our previous study showed that lactose can greatly enhance EPS yields in SM-A87, while other mono- and disaccharides cannot [10]. In the lactic acid bacterium *Bifidobacterium longum* subsp. *longum* CRC 002, lactose is a better sugar source for the production of EPS than glucose, galactose and fructose [14]. Lactose metabolism has an effect on EPS production in some other lactic acid bacteria [15,16]. These results indicate that lactose is an important nutritional factor that can affect bacterial EPS production. The aim of this study is to investigate how only lactose can stimulate EPS production in SM-A87. This work lays the foundation for further understanding the regulation of bacterial EPS production.

## Materials and Methods

### Carbon source utilization and EPS yield under different conditions

The bacterial strain SM-A87 is routinely maintained in our laboratory. Carbon source oxidization by SM-A87 was tested using the API 50 CH system (bioMerieux, Craponne, France) following the manufacturer’s instructions.

The basal medium contained 8.9 g peptone, 5.0 g yeast extract and 1 L artificial seawater at pH 8.0. EPS production was investigated by supplementing the basal medium with various carbon sources, including lactose, glucose, galactose and as well as combinations of glucose with either lactose or galactose. For the single carbon sources, the lactose concentration was 32.2 g/L (≈ 0.9 mol/L), which was the best concentration for SM-A87 EPS production [10]. The glucose and galactose concentration were all 1.8 mol/L, which had equivalent molarity of monosaccharide with supplied lactose. For the combinations, the concentration of the monosaccharide of each carbon source was 0.9 mol/L. To investigate the effect of isopropylthiogalactoside (IPTG) on EPS production, IPTG was added to the medium at a final concentration of 2 mM when SM-A87 reached an OD_600_ of approximately 0.6. SM-A87 was inoculated in different media and incubated at 10°C and 200 rpm for 6 days. Then, the EPS concentration was determined using the phenol-sulfuric acid method [17]. In the confirmation experiment, sucrose and galactose were added to the basal medium, and the IPTG concentration, culture conditions and EPS determination method were identical to those described above except that EPS concentration was determined at different time intervals. All experiments were independently repeated at least twice and performed in triplicate.

### RNA sequencing

For transcriptome analyses, SM-A87 was cultured at 10°C in basal medium with and without 32.2 g/L lactose. Medium supplemented with lactose is optimal for EPS production. EPS production began to increase sharply at day 4; EPS yields were near their highest at day 6 [10] (S1 Fig.). Therefore, days 4 and 6 were selected as the time points for transcriptome sequencing. On days 4 and 6, SM-A87 grown in both media was collected by centrifugation, and total RNA was isolated from RNA-stabilized bacteria with the RNeasy Protect Bacteria Mini Kit (Qiagene, Germany). RNA quality and concentration were measured using an RNA Pico chip on an Agilent Bioanalyzer. The rRNA depletion, mRNA enrichment, reverse transcription and cDNA sequencing were performed by the Beijing Genomics Institute (BGI, China). The cDNA libraries were sequenced using an Illumina HiSeq2000 instrument with reads length of 90 bp.

### Bioinformatics analyses

After clean sequencing data were obtained, the reads were mapped to the SM-A87 reference genome (accession no. CP001650) with SOAP2 [18]. Gene expression was normalized using the RPKM method (reads per kilobase of exon model per million mapped reads), and the RPKM value of each gene was used to compare its expression variation under different conditions [19]. The p value of a differentially expressed gene was calculated according to the method of Audic and Claverie, and the p value was adjusted using the FDR (false discovery rate) control method [20,21]. A gene was considered to be differentially regulated between two conditions when the gene showed a >2-fold absolute fold-change ratio and displayed an FDR-adjusted p value <0.01. The changes in the expression of overall lipopolysaccharide (LPS) and of the EPS gene clusters were tested using the T-profiler method [22].

The DNA binding sites of the LacI transcriptional regulator on the SM-A87 genome were predicted by the PePPER webserver using the motif mining method (http://pepper.molgenrug.nl) [23]. This webserver can also predict whether two genes are in the same operon. The metabolic pathway of SM-A87 was predicted by the Kyoto Encyclopedia of Genes and Genomes (KEGG) website [24]. The complete genome sequence of SM-A87 has already been published, and the metabolic pathway has been analyzed by KEGG. The genes of SM-A87 that are associated with KEGG pathways were directly obtained from the KEGG pathway maps.

### Real-time quantitative PCR (RT-qPCR)

SM-A87 was cultured in the basal medium, basal medium supplied with lactose as well as combination of glucose and galactose. Strains were collected by centrifugation at days 3, 4 and 6. When SM-A87 grown to OD_600_ ≈ 0.6 in the basal medium supplied with sucrose and galactose, the IPTG was added into the medium at a final concentration of 2 mM. Strains cultured with and without IPTG were collected 24 h and 42 h after the supplement of IPTG. The total RNA was extracted from these collected cells as described in the RNA sequencing. Reverse transcription was performed using the PrimeScript RT reagent Kit with gDNA Eraser (Perfect Real Time) (TaKaRa, Japan). The obtained cDNA and the SYBR *Premix Ex Taq* (Tli RNaseH Plus) (TaKaRa, Japan) were used to prepare the quantitative PCR reaction system, and the reaction was accomplished with the LightCycler 480 (Roche). Fold change of the target gene was determined using the comparative threshold cycle method by comparison to the rpoD housekeeping gene of SM-A87.

### RNA-seq data accession number

The RNA-seq reads data of four samples have been deposited in NCBI’s sequence read archive (SRA) under accession number SRA098107.

## Results

### Carbon source utilization and EPS production of SM-A87 in different media

**Fig 1:**
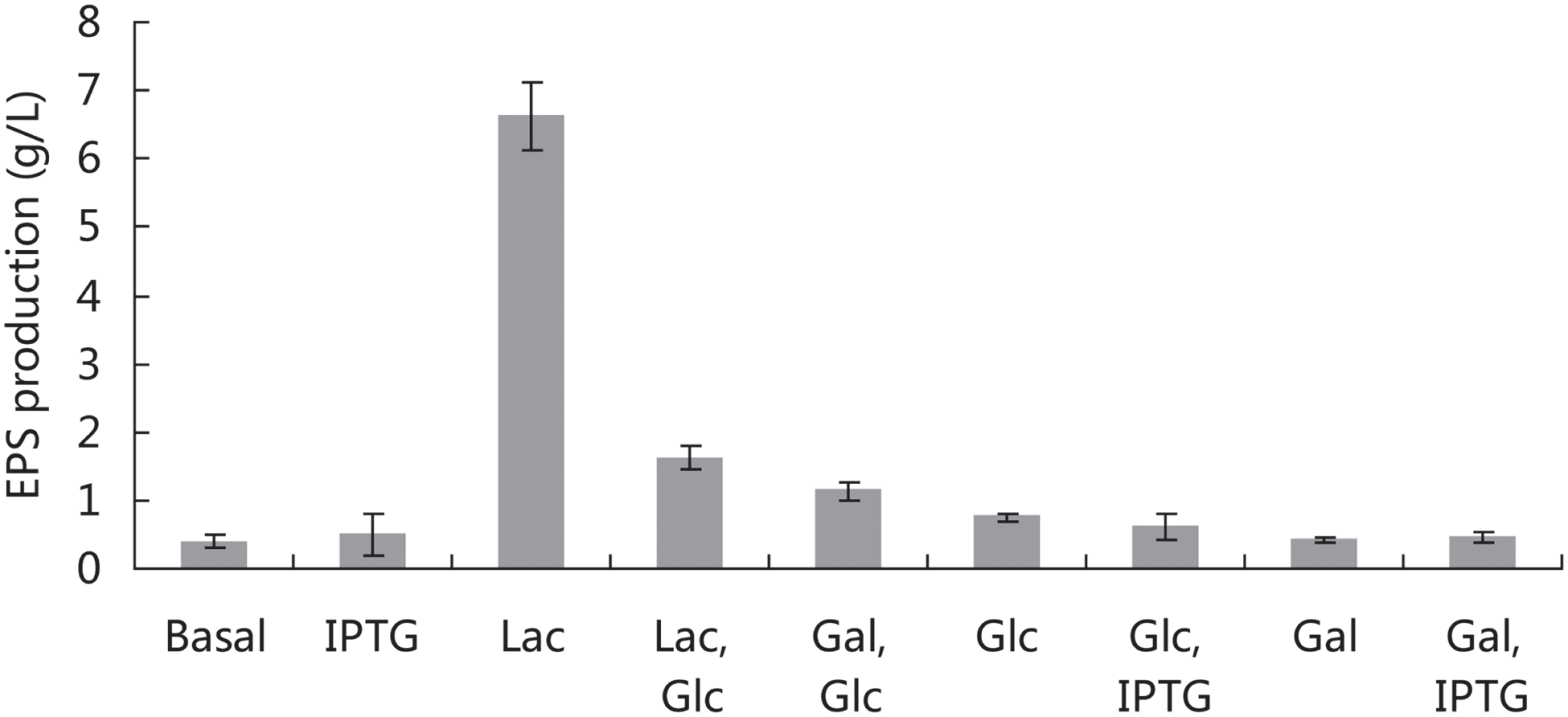
Effect of carbon sources and IPTG on EPS production in strain SM-A87. Abbreviations: Basal, basal medium without extra carbon sources; IPTG, isopropylthiogalactoside; Lac, lactose; Glc, glucose, Gal, galactose.

SM-A87 could oxidize glycerol, L-arabinose, D-xylose, galactose, glucose, fructose, mannose, maltose, fucose, lactose and sucrose according to the API 50CH test. Our previous study showed that lactose was the optimal carbon source for EPS production in SM-A87 and had significant advantages over the other tested carbon sources, including glucose, mannose, maltose and sucrose [10]. The results in this study showed that lactose significantly promoted EPS production in SM-A87 compared with galactose and combinations of either glucose and lactose or glucose and galactose. The addition of IPTG had no significant effect on EPS production in different media (Fig. 1). When lactose was the carbon source, the EPS production was 6.6 g/L. In other cases, the EPS production was less than 2.0 g/L. When the medium contained lactose and glucose simultaneously, the EPS production was also low (1.6 g/L), which indicated that glucose could inhibit the enhancement effects of lactose on EPS production in SM-A87. When EPS production increased sharply from day 4 to day 6, the lactose concentration in the medium decreased sharply, which implied that the lactose was consumed by SM-A87 to produce EPS (S1 Fig.). The biomass of SM-A87 grew in the medium with lactose as the sole carbon source can reach a high value (S2 Fig.). This indicated that lactose can stimulate bacterial growth and high biomass was positively correlated with high EPS production.

### The transcriptomes of SM-A87 under different culture conditions

RNA-seq (direct cDNA sequencing) allows bacterial transcription to be studied at a high resolution. This technique is now widely used in bacterial transcriptome studies [25,26,27,28,29,30]. High-throughput RNA-seq technology was used to study the transcriptional landscape of SM-A87 under different culture conditions. To perform a detailed analysis of lactose-regulated genes, we compared the expression profiles of SM-A87 grown in the basal medium with or without lactose at days 4 (sample name: L4, with lactose; O4, without lactose) and 6 (L6, with lactose; O6, without lactose). The sequencing throughput data and the number of reads aligned on the reference genome for the four samples are shown in Table 1. For each sample, more than 6.4 million 90-bp reads were produced and more than 96% of the reads could be aligned to the reference genome. The percentage of reads uniquely mapped to the coding sequence (CDS) region was between 69% and 75%, which indicated that the rRNA depletion during the sample preparation was highly efficient.

**Table 1:** Summary of RNA-seq data for each sample.

Sample	Total no. of reads	Reads mapped to reference genome	Percentage^a^	Reads uniquely mapped to CDS	Percentage^b^
O4	6,562,170	6,365,032	97%	4,847,982	74%
O6	6,454,846	6,225,063	96%	4,428,575	69%
L4	6,917,370	6,703,707	97%	5,160,919	75%
L6	6,426,822	6,259,141	97%	4,845,073	75%

^a^, Percentage of reads mapped to reference genome

^b^, Percentage of reads uniquely mapped to CDS

We were mainly concerned with the up-regulated genes induced by lactose. On day 4, 10.9% of the SM-A87 genes (507 out of 4653) were expressed more than twice as much (p < 0.01) during growth in the lactose medium, while 10.8% of the genes (504 out of 4653) were induced two-fold or higher (p < 0.01) during growth in the lactose medium on day 6. Though the number of up-regulated genes at the two time points was almost equal, the gene sets were different. These two time points only shared 211 up-regulated genes. On day 4, the up-regulated gene sets were enriched in genes encoding proteins involved in clusters of orthologous groups (COGs) with functions related to carbohydrate transport and metabolism [G] and translation, ribosomal structure and biogenesis [J] (S3 Fig.). By day 6, a higher proportion of up-regulated genes were involved in COGs with functions related to replication, recombination and repair [L], cell wall/membrane/envelope biogenesis [M] and inorganic ion transport and metabolism [P] (S3 Fig.). This result indicated that there were different transcriptional regulation strategies at different stages of the SM-A87 EPS production process.

### The expression of genes within the EPS biosynthesis gene cluster was up-regulated when lactose was added

Polysaccharide biosynthesis requires enzymes involved in the formation of activated nucleotide sugar precursors, glycosyltransferases, proteins involved in the transport of the repeat unit across the cytoplasmic membrane and enzymes involved in EPS polymerization and secretion [31,32,33]. The genes encoding these proteins are usually grouped together to form a polysaccharide biosynthetic gene cluster [34,35,36]. A genomic study showed that SM-A87 has two gene clusters involved in polysaccharide synthesis and export [37]. One cluster containing 25 ORFs (ZPR_0543 to ZPR_0567) is assumed to be responsible for EPS synthesis because several ORFs in this cluster have sequence similarity to proteins, such as Wza, Wzc and Wzi, that are involved in capsular polysaccharide biosynthesis in *Escherichia coli* [32,38]. This predicted EPS gene cluster is 25 kbp long, and all the ORFs except ZPR_0564 are in the same orientation. ORF ZPR_0564 is only 117 bp long and encodes a hypothetical protein. This gene may have been predicted incorrectly and is excluded from the EPS gene cluster. Consistent with this result, no RNA-seq reads could be aligned to the ORF ZPR_0564 sequence. The blastp search in the GenBank database showed that ZPR_0564 had no reliable homolog in any other organism. This result further confirmed that this gene was predicted incorrectly. Thus, the EPS biosynthetic gene cluster contains a total of 24 genes, which are listed in Table 2.

**Table 2:** Expression level of genes within the EPS gene cluster^a^.

Locus tag	Annotation	Scale of expression level	
		L4/O4	L6/O6
ZPR_0543	UDP-glucose 6-dehydrogenase	2.6	2.2
ZPR_0544	GDP-fucose synthetase	1.6	2.6
ZPR_0545	GDP-D-mannose dehydratase	1.7	2.0
ZPR_0546	polysaccharide biosynthesis protein	1.8	2.0
ZPR_0547	hypothetical protein	1.3	1.8
ZPR_0548	galactoside acetyltransferase (lacA)	-	2.9
ZPR_0549	hypothetical protein	1.9	1.8
ZPR_0550	acetyltransferase	1.9	1.7
ZPR_0551	glycosyl transferase	1.5	2.4
ZPR_0552	membrane protein	-	2.0
ZPR_0553	glycosyl transferase	-	2.1
ZPR_0554	hypothetical protein	1.4	4.0
ZPR_0555	trimeric LpxA-like enzyme	-	2.1
ZPR_0556	glycosyl transferase	-	3.2
ZPR_0557	glycosyl transferase	1.6	3.3
ZPR_0558	WfeP	1.4	2.0
ZPR_0559	4Fe-4S ferredoxin iron-sulfur binding protein	-	2.5
ZPR_0560	transferase	-	1.8
ZPR_0561	glycosyl transferase	1.3	2.6
ZPR_0562	putative dNTP-hexose dehydratase-epimerase	1.7	3.9
ZPR_0563	glycosyl transferase	2.3	1.2
ZPR_0565	glycosyltransferase	1.3	1.7
ZPR_0566	polysaccharide export protein	1.6	2.1
ZPR_0567	tyrosine-protein kinase ptk	1.5	2.0

^a^, ‘-’ denotes that the change of expression level is not significant.

The expression levels of the ORFs within the EPS cluster were compared when SM-A87 was cultured in the media with and without lactose (Table 2). At day 4, only 2 genes were up-regulated two-fold or higher (p<0.01) when SM-A87 was cultured in the medium with lactose compared with the control (without lactose). At day 6, however, 18 genes were up-regulated two-fold or higher (p<0.01). Of the up-regulated genes at day 6, ZPR_0543 encodes a UDP-glucose 6-dehydrogenase that can convert UDP-glucose to UDP-glucuronate. ZPR_0544 encodes a GDP-fucose synthetase, and ZPR_0545 encodes a GDP-D-mannose dehydratase that can convert GDP-mannose to GDP-fucose. Five of the eight glycosyl transferase genes in the EPS gene cluster were up-regulated two-fold or higher at day 6. Polysaccharide biosynthesis and export proteins (ZPR_0546, ZPR_0566 and ZPR_0567) were also up-regulated at day 6. Tested with the T-profiler method, the EPS gene group had a higher mean expression level than that of all of the other genes in the SM-A87 genome at days 4 and 6 (p<0.02 and p<0.001 for days 4 and 6, respectively). The RT-qPCR results confirmed that the expression levels of ORFs ZPR_0544, ZPR_0546, ZPR_0558 and ZPR_0566 were up-regulated in the lactose medium. As a negative control, when SM-A87 was cultured in the medium with glucose and galactose, the EPS production was low and the expression levels of these four genes within this EPS gene cluster were down-regulated or changed not statistically (S4 Fig.). This result indicated that, when the EPS yield was high, the expression level of EPS genes was up-regulated, implying that the expression level of genes within the EPS gene cluster was regulated when lactose was present in the medium.

### Lactose does not regulate the expression of genes within the lipopolysaccharide biosynthesis gene cluster

Another polysaccharide synthesis gene cluster containing ORFs from ZPR_1091 to ZPR_1126 is predicted to be involved in lipopolysaccharide (LPS) synthesis because two ORFs (ZPR_1094, ZPR_1095) encode an ABC transporter involved in LPS transport. Moreover, one ORF (ZPR_1123) encodes an O-antigen polymerase that is located in this cluster. The majority of the LPS genes did not change their expression levels in the two media (S1 Table). On day 4, only 3 genes were up-regulated, and 1 gene was down-regulated two-fold or higher (p<0.01) in the medium with lactose. On day 6, no gene was up-regulated, and 4 genes were down-regulated two-fold or higher (p<0.01). Therefore, there was no obvious variation in the expression profiles of the LPS gene cluster at different stages when SM-A87 was grown in different media. This result indicates that lactose could not regulate LPS genes and that the LPS gene cluster was not related to EPS production in SM-A87. Consistent with this conclusion, the T-profiler t-test showed that the mean expression level of the LPS genes group was not significantly different from that of all other genes at days 4 and 6 (p>0.1 for two time points).

### Other genes for EPS synthesis outside the EPS gene cluster

Most of the genes involved in EPS synthesis are usually grouped to form a gene cluster. However, the gene cluster does not encompass all of the genes responsible for EPS synthesis. Some functional genes are dispersed in the genome, such as the genes responsible for the formation of some nucleotide sugar precursors that provide elements of oligosaccharide units. The expression levels of these genes were compared when SM-A87 was cultured in different media (Table 3). The genes related to galactose precursor synthesis were up-regulated at days 4 and 6, which is consistent with the addition of lactose, which contains galactoside. The RT-qPCR results showed that the expression level of ORF ZPR_2833 was up-regulated when the media containing lactose or galactose (S4 Fig.). ORF ZPR_2582, which is responsible for converting glucose to glucose-6-phosphate, was down-regulated more than two-fold. This was confirmed by RT-qPCR analysis of this gene. The expression levels of other genes related to the conversion of glucose, fructose and mannose changed less than two-fold. These genes encode enzymes involved in many other cellular metabolic processes; therefore, their expression regulation is complex and is less related to lactose metabolism. ORF ZPR_0538 encodes a mannose-1-phosphate guanylyltransferase that can convert mannose-1-phosphate to the precursor GDP-mannose. This ORF was up-regulated 2.4- and 1.9-fold at days 4 and 6, respectively. These analyses indicated that lactose can also up-regulate genes related to EPS synthesis that are located outside the EPS gene cluster.

**Table 3:** Expression level and function of EPS synthesis genes outside the EPS gene cluster.

Locus tag	Annotation	Function	Scale of expression level	
			L4/O4	L6/O6
ZPR_2833	galactokinase	convert galactose to galactose-1-p	12.9	22.3
ZPR_2834	galactose-1-phosphate uridylyltransferas	convert galactose-1-p to UDP-galactose	15.0	51.3
ZPR_2610	UDP-glucose 4-epimerase	convert UDP-galactose to UDP-glucose	1.2	2.3
ZPR_2582	carbohydrate kinase	convert glucose to glucose-6-p	0.4	0.5
ZPR_0194	glucose-6-phosphate isomerase	convert glucose-6-p to fructose-6-p	0.5	1.2
ZPR_2216	phosphomannose isomerase	convert fructose-6-p to mannose-6-p	1.2	1.5
ZPR_1735	phosphomannomutase	convert mannose-6-p to mannose-1-p	0.7	0.8
ZPR_0538	mannose-1-phosphate guanylyltransferase	convert mannose-1-p to GDP-mannose	2.4	1.9

## Discussion

Many factors can influence bacterial EPS production [2,3], the regulatory mechanisms of which are infrequently studied. In SM-A87, lactose can greatly promote EPS production, while other sugars cannot. SM-A87 can utilize a variety of sugars, which indicates that the ability of SM-A87 to use different sugar sources cannot explain the observed differences in the EPS yield. Lactose promotion of EPS production in SM-A87 can be dramatically inhibited by glucose (Fig. 1). Notably, glucose transport and metabolism can inhibit the uptake of lactose and other carbohydrates [39, 40]. This effect strongly implies that EPS production in SM-A87 may be regulated in a manner similar to lactose utilization in *E. coli*, where lactose regulates the expression of several genes on the lactose operon. The expression of the genes within the lactose operon can be induced by lactose, and *E. coli* can then utilize the lactose. When glucose is present, the expression of genes within the lactose operon is repressed, and lactose is not utilized [41]. Similarly, in SM-A87, lactose alone can promote EPS production significantly, while the presence of glucose can inhibit this effect. Supplement of glucose and galactose simultaneously into the culture medium can not promote EPS production, showing that lactose must be transported into the cell to commit promotion function. The SM-A87 genome contains beta-galactosidase genes but no lactose operon. Therefore, the genes regulated by lactose in SM-A87 would be different from those in *E. coli*. For example, the SM-A87 genome contains five annotated beta-galactosidases. However, only one beta-galactosidase gene (ZPR_4170) was up-regulated 2.1-fold at day 4 when lactose was present. Transcriptome analyses showed that lactose could up-regulate the genes related to EPS synthesis in SM-A87, especially those in the EPS gene cluster. Gene regulation by lactose in *E. coli* requires the LacI transcriptional regulator, which can suppress gene expression and be deactivated by lactose [41]. The SM-A87 genome harbors three genes encoding the LacI family transcriptional regulator (ZPR_2255, ZPR_2821 and ZPR_2902). Notably, the expression of gene ZPR_2255 was up-regulated at both days 4 and 6. A prediction of transcriptional regulator binding sites showed that LacI could bind the upstream sequences of ORFs ZPR_0546 and ZPR_0558, which are within the EPS gene cluster. The PePPER webserver predicts that ORFs ZPR_0546 to ZPR_0554 are within the same operon and that ORFs ZPR_0558 to ZPR_0567 are within another operon. The expression levels of these two operons were up-regulated when lactose was added to the basal medium. This up-regulation implies that the regulation of EPS gene expression by lactose may depend on the activity of the LacI transcriptional regulator. However, transcriptional regulation in bacteria is complex. Whether LacI regulates the EPS gene cluster with lactose needs to be further confirmed experimentally.

IPTG is an analogue of lactose and can be used as an inducer to regulate gene expression. It is strange that the addition of IPTG to the medium cannot promote EPS production in SM-A87 grown with galactose and glycerol as the carbon source. The glycosyl composition of the EPS secreted by SM-A87 has been analyzed by GC/MS, and the major components are glucose (36.5%), mannose (30.7%) and galactose (10.5%) [13]. Based on the KEGG metabolic pathway analyses, precursor GDP-mannose can be converted from glucose in SM-A87 through the functions of proteins ZPR_0538, ZPR_0194, ZPR_1735, ZPR_2216 and ZPR_2582 (Table 3). Therefore, it is conceivable that the strain might require a large amount of glucose for EPS production. However, SM-A87 lacks the gene necessary to convert glucose-6-phosphate to UDP-glucose, while it contains the genes (ZPR_2610, galE) for converting UDP-galactose to UDP-glucose. This analysis indicates that the precursor UDP-glucose cannot be synthesized from glucose and must therefore be synthesized from galactose. As a result, SM-A87 requires large amounts of both glucose and galactose for high EPS production. Lactose provides these two sugars simultaneously and avoids glucose inhibition. Therefore, lactose is the best sugar source for EPS production, and IPTG cannot improve EPS production when only one sugar is present in the medium. In addition, lactose can greatly stimulate the growth of SM-A87, showing that lactose also provides proper sugar for bacterial growth which is an advantage for EPS production.

**Fig 2:**
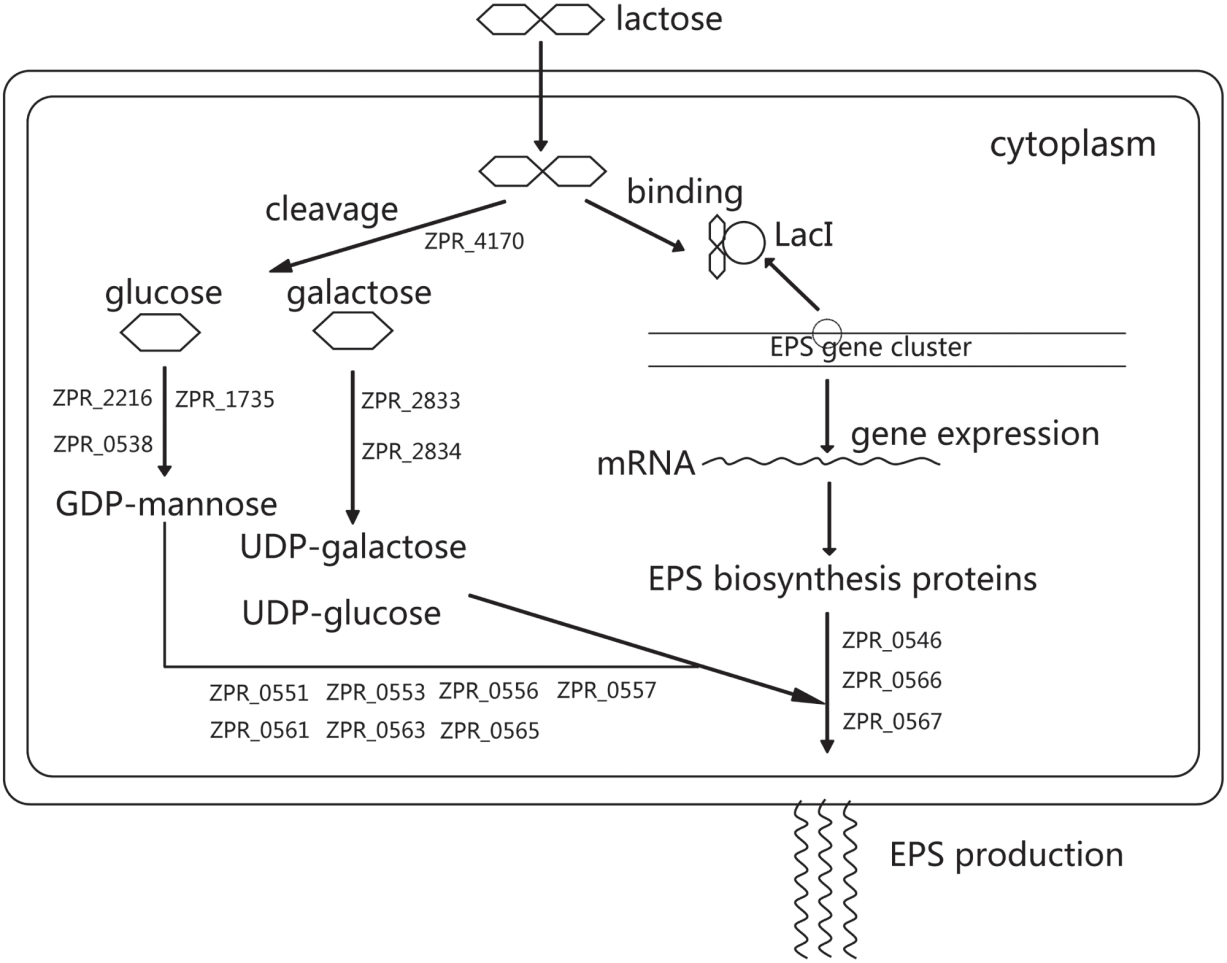
A predicted schematic diagram to illustrate how lactose improves EPS production in SM-A87. The EPS synthetic genes up-regulated by lactose were mapped onto this diagram. ZPR_4170, beta-galactosidase; ZPR_2216, phosphomannose isomerase; ZPR_1735, phosphomannomutase; ZPR_0538, mannose-1-phosphate guanylyltransferase; ZPR_2833, galactokinase; ZPR_2834, galactose-1-phosphate uridylyltransferase; ZPR_0551, ZPR_0553, ZPR_0556, ZPR_0557, ZPR_0561, ZPR_0563, ZPR_0565, glycosyl transferase; ZPR_0546, ZPR_0566, ZPR_0567, polysaccharide biosynthesis and export protein.

**Fig 3:**
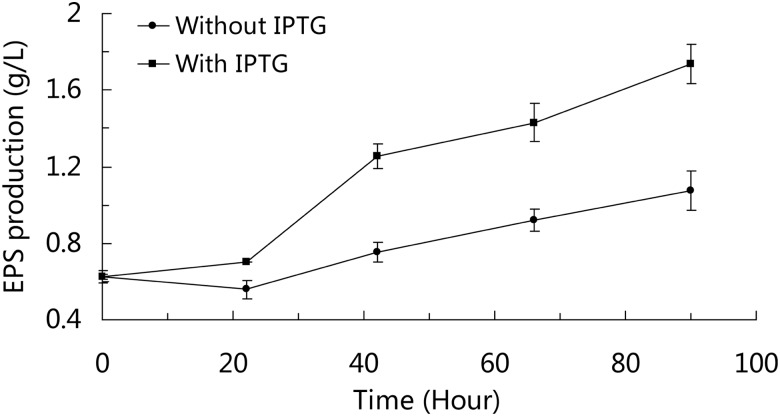
EPS production curve of SM-A87 induced with and without IPTG. Strains were grown in the basal medium supplemented with sucrose and galactose. The differences in EPS production at 42, 66 and 90 hours are statistically significant (p < 0.01, two samples t-test).

How lactose improves EPS production in SM-A87 was summarized in Fig. 2, in which the problem that lactose regulates EPS synthesis genes directly or through metabolites of lactose is still unclear based on our data and needs to be further studied. It is interesting that when SM-A87 grown with combination of glucose and galactose, the expression levels of EPS-related genes within the EPS cluster were slightly improved during the early days of the fermentation, which resembles the effect of lactose (S4 Fig.). This implies that lactose may regulate the EPS synthesis through other metabolites in the late days of the fermentation when the EPS production was high. Lactose can greatly improve the growth of SM-A87, which could also contribute to the high EPS yield. To confirm the behavior that lactose can not only act as an inducer but also provide perfect nutrients for EPS production in SM-A87, we designed a medium containing sucrose and galactose that could simulate the nutritional composition of lactose. Subsequently, IPTG was added as an inducer. If the above hypothesis is correct, then the EPS production should be improved when the medium is supplemented with IPTG. The experimental results showed that the EPS production was higher when the medium was supplemented with IPTG (p<0.01) (Fig. 3). The RT-qPCR analyses showed that genes within the EPS gene cluster were up-regulated when IPTG was present in the medium (S5 Fig.). These results supported the conclusion that high EPS yield in SM-A87 requires both an inducer and the proper nutrients. This work provides a better understanding of the regulation of ecologically important EPS production in marine bacteria.

SM-A87 was isolated from sediment in the southern Okinawa Trough, an important depocenter of organic matter from the East China shelf, which itself is adjacent to the Changjiang River mouth. Every year, a large amount of organic carbon is exported from the shelf to the southern Okinawa Trough [42]. This behavior is consistent with the fact that SM-A87 can utilize a variety of carbon sources. The detailed composition of the deposited organic carbon in the southern Okinawa Trough is not clear. However, as the organic carbon was transferred from both the shelf and the continent of Asia, the organic carbon deposited in the southern Okinawa Trough may contain lactose and thus might stimulate EPS production in SM-A87. SM-A87 may benefit from the production of EPS, which can concentrate organic matter and metal ions. For example, at day 6, when the EPS yield reached its maximum, the genes encoding the iron complex ABC transport (ZPR_0399, ZPR_0400 and ZPR_0401) were up-regulated. These proteins can transport the iron ions concentrated by EPS into the cell, which would be an advantage for the survival of SM-A87 in the sediment.

## Supporting Information

S1 Fig The fermentation process of strain SM-A87 cultured under optimum culture conditions. EPS production, residual lactose in the medium and cell growth were measured against time. The culture medium consisted of 32.2 g/L lactose, 8.87 g/L peptone and 5 g/L yeast extract.
(DOC)

S2 Fig The growth curves of strain SM-A87 cultured in media supplied with different carbon sources. The abbreviations are described in Fig. 1.
(DOC)

S3 Fig Comparison of COG functions of genes up-regulated at days 4 and 6. COG functional categories: Energy production and conversion [C]; Amino acid transport and metabolism [E]; Nucleotide transport and metabolism [F]; Carbohydrate transport and metabolism [G]; Coenzyme transport and metabolism [H]; Lipid transport and metabolism [I]; Translation, ribosomal structure and biogenesis [J]; Transcription [K]; Replication, recombination and repair [L]; Cell wall/membrane/envelope biogenesis [M]; Posttranslational modification, protein turnover, chaperones [O]; Inorganic ion transport and metabolism [P]; Secondary metabolite biosynthesis, transport and catabolism [Q]; General function prediction only [R]; Function unknown [S]; Signal transduction mechanisms [T]; Intracellular trafficking, secretion, and vesicular transport [U]; Defense mechanisms [V].
(DOC)

S4 Fig The differential expression of target genes of SM-A87 cultured in different media at days 2, 4 and 6 as determined by RT-qPCR. The control is basal medium. (A) Basal medium supplied with lactose; (B) basal medium supplied with combination of glucose and galactose. The ORF ZPR_2833 is galactokinase; ORFs ZPR_0544, ZPR_0546, ZPR_0558 and ZPR_0566 are EPS biosynthesis genes within the EPS gene cluster; ORF ZPR_1094 is LPS related gene; ORF ZPR_2582 is carbohydrate kinase.
(DOC)

S5 Fig The differential expression of target genes of SM-A87 induced with IPTG as determined by RT-qPCR. Strains were grown in the basal medium supplemented with sucrose and galactose. After 5 and 42 hours of supplement of IPTG, the cells were collected and RT-qPCR experiments were performed.
(DOC)

S1 Table Expression level of genes within the LPS gene cluster. (DOC)
