# Supplementary material for: Comparative Transcriptome Analysis Reveals That Lactose Acts as an Inducer and Provides Proper Carbon Sources for Enhancing Exopolysaccharide Yield in the Deep-Sea Bacterium Zunongwangia profunda SM-A87
Source: PLoS One. 2015 Feb 13;10(2):e0115998. doi: 10.1371/journal.pone.0115998 (PMC4332637; doi:10.1371/journal.pone.0115998)
Supplement: S1 Fig — EPS production, residual lactose in the medium and cell growth were measured against time. The culture medium consisted of 32.2 g/L lactose, 8.87 g/L peptone and 5 g/L yeast extract. (DOC) [file pone.0115998.s001.doc]

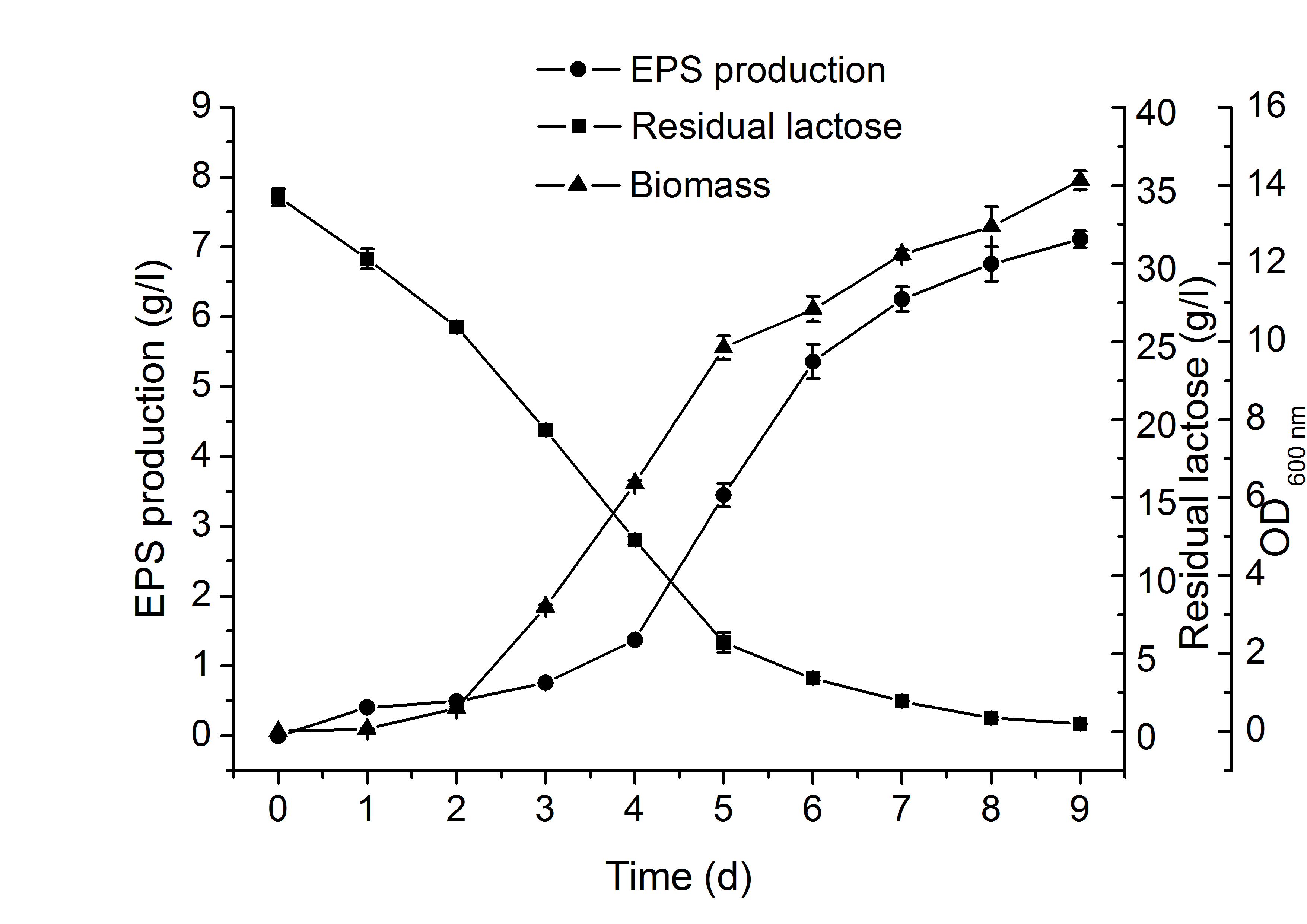


**Figure S1. The fermentation process of strain SM-A87 cultured under the optimum culture conditions.** The EPS production, residual lactose in the medium and cell growth were detected with time. The culture medium consisted of 32.2 g/L lactose, 8.87 g/L peptone, 5 g/L yeast extract.
