# Supplementary material for: Comparative Transcriptome Analysis Reveals That Lactose Acts as an Inducer and Provides Proper Carbon Sources for Enhancing Exopolysaccharide Yield in the Deep-Sea Bacterium Zunongwangia profunda SM-A87
Source: PLoS One. 2015 Feb 13;10(2):e0115998. doi: 10.1371/journal.pone.0115998 (PMC4332637; doi:10.1371/journal.pone.0115998)
Supplement: S2 Fig — The abbreviations are described in Fig. 1. (DOC) [file pone.0115998.s002.doc]

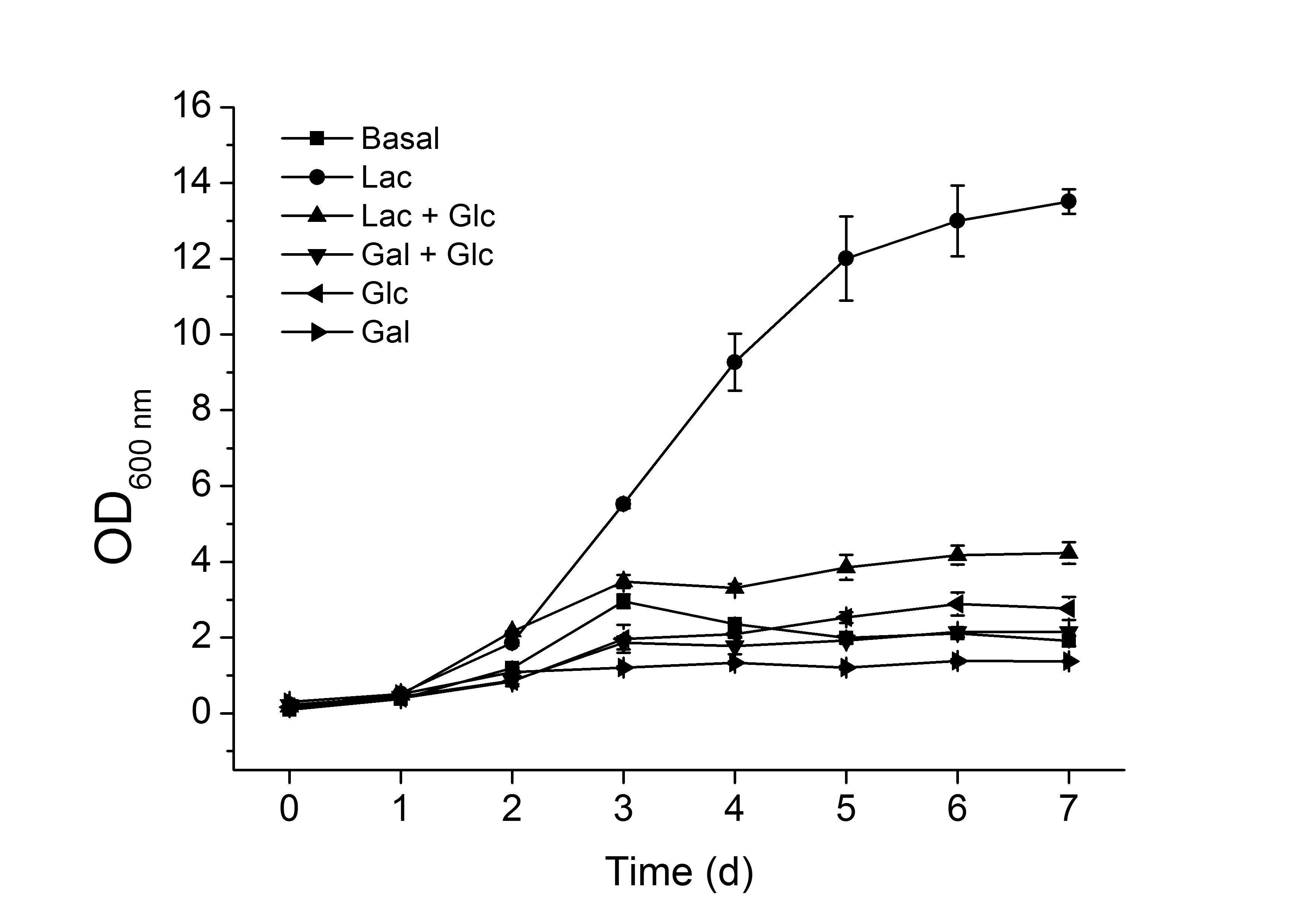


**Figure S2. The growth curves of strain SM-A87 cultured in media supplied with different carbon sources.** The abbreviations are described in Figure 1.
