# Supplementary material for: Comparative Transcriptome Analysis Reveals That Lactose Acts as an Inducer and Provides Proper Carbon Sources for Enhancing Exopolysaccharide Yield in the Deep-Sea Bacterium Zunongwangia profunda SM-A87
Source: PLoS One. 2015 Feb 13;10(2):e0115998. doi: 10.1371/journal.pone.0115998 (PMC4332637; doi:10.1371/journal.pone.0115998)
Supplement: S3 Fig — COG functional categories: Energy production and conversion [C]; Amino acid transport and metabolism [E]; Nucleotide transport and metabolism [F]; Carbohydrate transport and metabolism [G]; Coenzyme transport and metabolism [H]; Lipid transport and metabolism [I]; Translation, ribosomal structure and biogenesis [J]; Transcription [K]; Replication, recombination and repair [L]; Cell wall/membrane/envelope biogenesis [M]; Posttranslational modification, protein turnover, chaperones [O]; Inorganic ion transport and metabolism [P]; Secondary metabolite biosynthesis, transport and catabolism [Q]; General function prediction only [R]; Function unknown [S]; Signal transduction mechanisms [T]; Intracellular trafficking, secretion, and vesicular transport [U]; Defense mechanisms [V]. (DOC) [file pone.0115998.s003.doc]

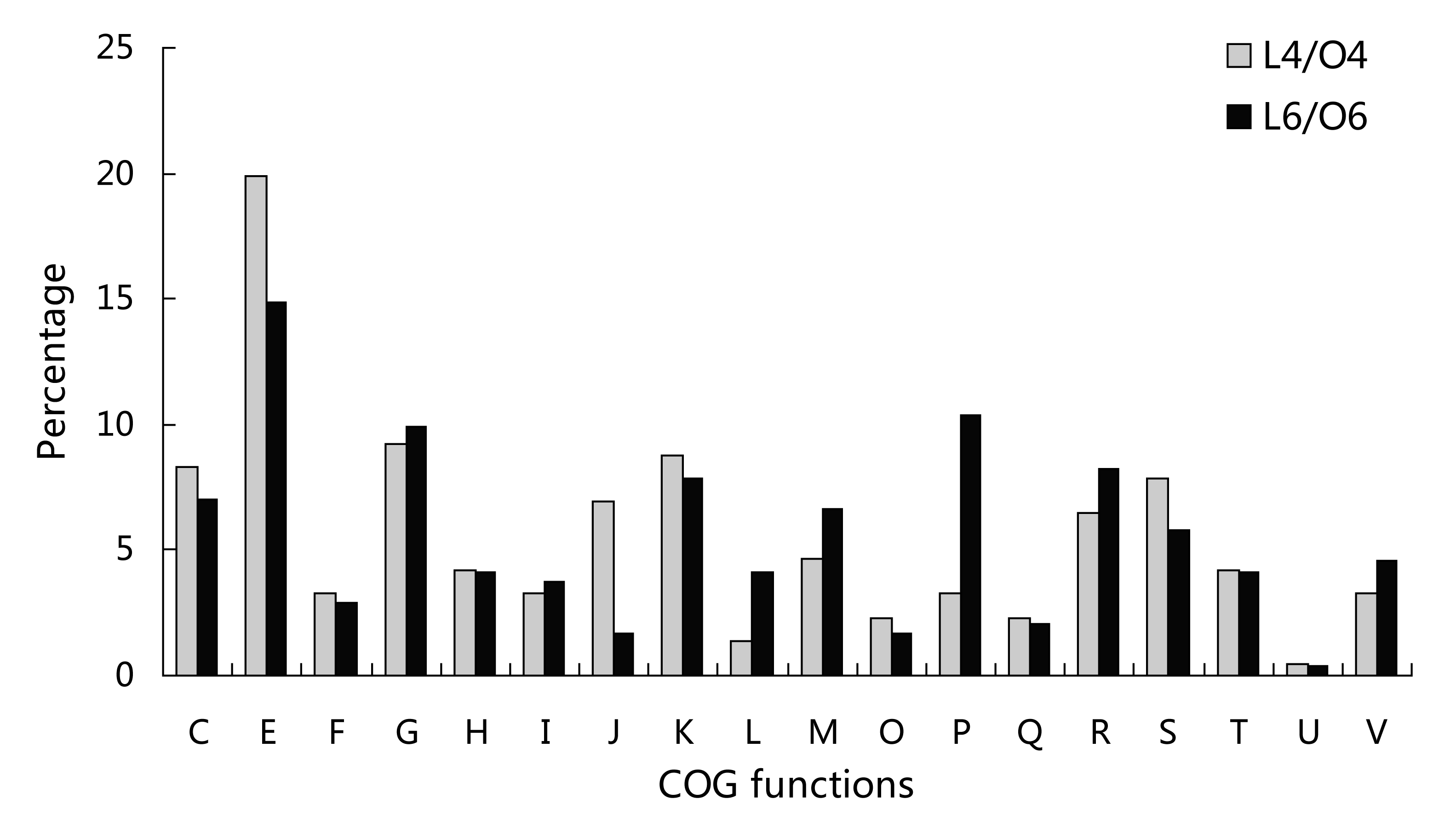


**Figure S3. Comparison of COG functions of genes up regulated at days 4 and 6.** COG functional categories: Energy production and conversion [C]; Amino acid transport and metabolism [E]; Nucleotide transport and metabolism [F]; Carbohydrate transport and metabolism [G]; Coenzyme transport and metabolism [H]; Lipid transport and metabolism [I]; Translation, ribosomal structure and biogenesis [J]; Transcription [K]; Replication, recombination and repair [L]; Cell wall/membrane/envelope biogenesis [M]; Posttranslational modification, protein turnover, chaperones [O]; Inorganic ion transport and metabolism [P]; Secondary metabolite biosynthesis, transport and catabolism [Q]; General function prediction only [R]; Function unknown [S]; Signal transduction mechanisms [T]; Intracellular trafficking, secretion, and vesicular transport [U]; Defense mechanisms [V].
