# Supplementary material for: Comparative Transcriptome Analysis Reveals That Lactose Acts as an Inducer and Provides Proper Carbon Sources for Enhancing Exopolysaccharide Yield in the Deep-Sea Bacterium Zunongwangia profunda SM-A87
Source: PLoS One. 2015 Feb 13;10(2):e0115998. doi: 10.1371/journal.pone.0115998 (PMC4332637; doi:10.1371/journal.pone.0115998)
Supplement: S4 Fig — The control is basal medium. (A) Basal medium supplied with lactose; (B) basal medium supplied with combination of glucose and galactose. The ORF ZPR_2833 is galactokinase; ORFs ZPR_0544, ZPR_0546, ZPR_0558 and ZPR_0566 are EPS biosynthesis genes within the EPS gene cluster; ORF ZPR_1094 is LPS related gene; ORF ZPR_2582 is carbohydrate kinase. (DOC) [file pone.0115998.s004.doc]

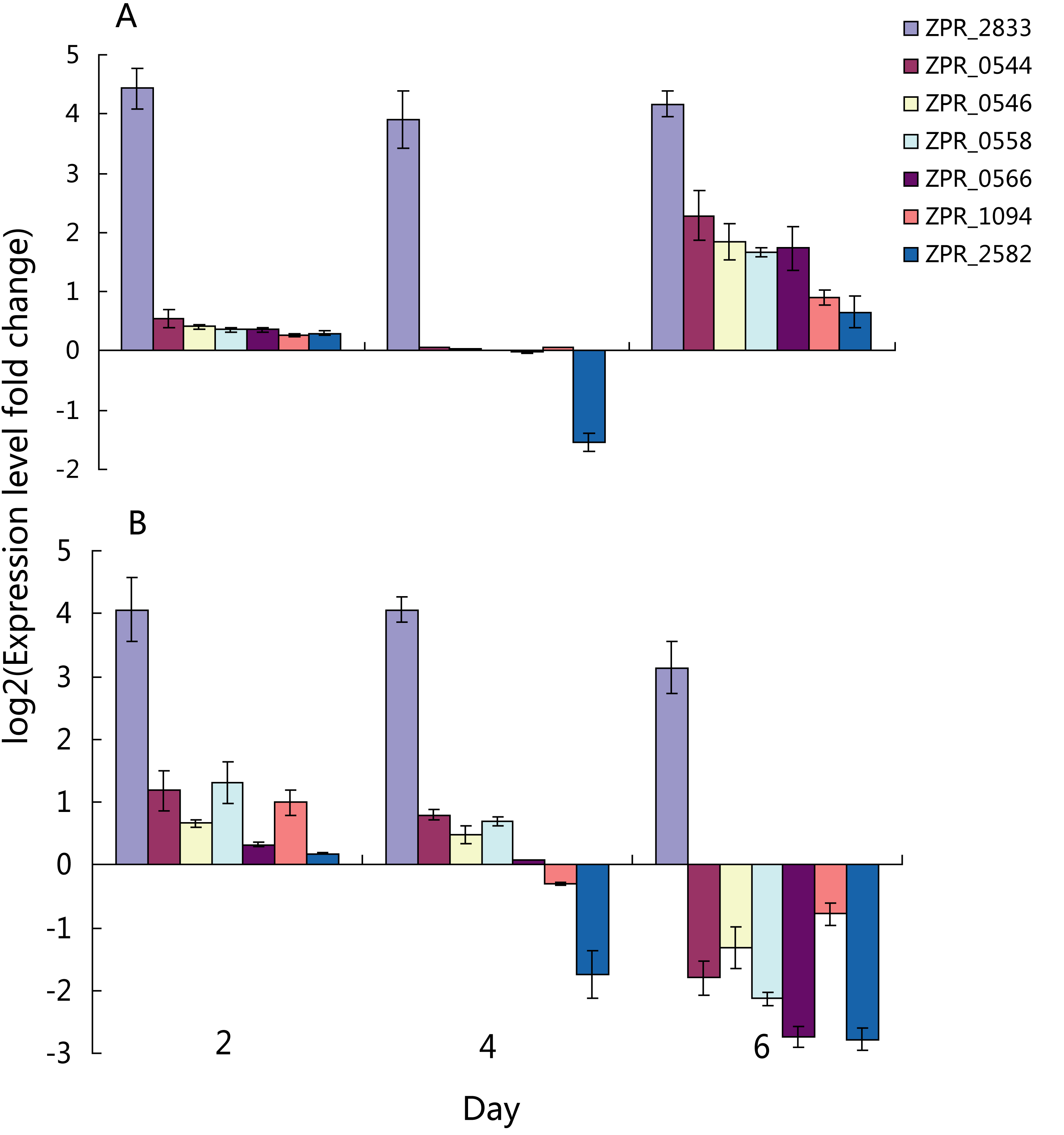


**Figure S4. The differential expression of target genes of SM-A87 cultured in different medias at days 2, 4 and 6 as determined by RT-qPCR.** The control is basal medium. (A) Basal medium supplied with lactose; (B) basal medium supplied with combination of glucose and galactose. The ORF ZPR_2833 is galactokinase; ORFs ZPR_0544, ZPR_0546, ZPR_0558 and ZPR_0566 are EPS biosynthesis genes within the EPS gene cluster; ORF ZPR_1094 is LPS related gene; ORF ZPR_2582 is carbohydrate kinase.
