# Supplementary material for: Comparative Transcriptome Analysis Reveals That Lactose Acts as an Inducer and Provides Proper Carbon Sources for Enhancing Exopolysaccharide Yield in the Deep-Sea Bacterium Zunongwangia profunda SM-A87
Source: PLoS One. 2015 Feb 13;10(2):e0115998. doi: 10.1371/journal.pone.0115998 (PMC4332637; doi:10.1371/journal.pone.0115998)
Supplement: S5 Fig — Strains were grown in the basal medium supplemented with sucrose and galactose. After 5 and 42 hours of supplement of IPTG, the cells were collected and RT-qPCR experiments were performed. (DOC) [file pone.0115998.s005.doc]

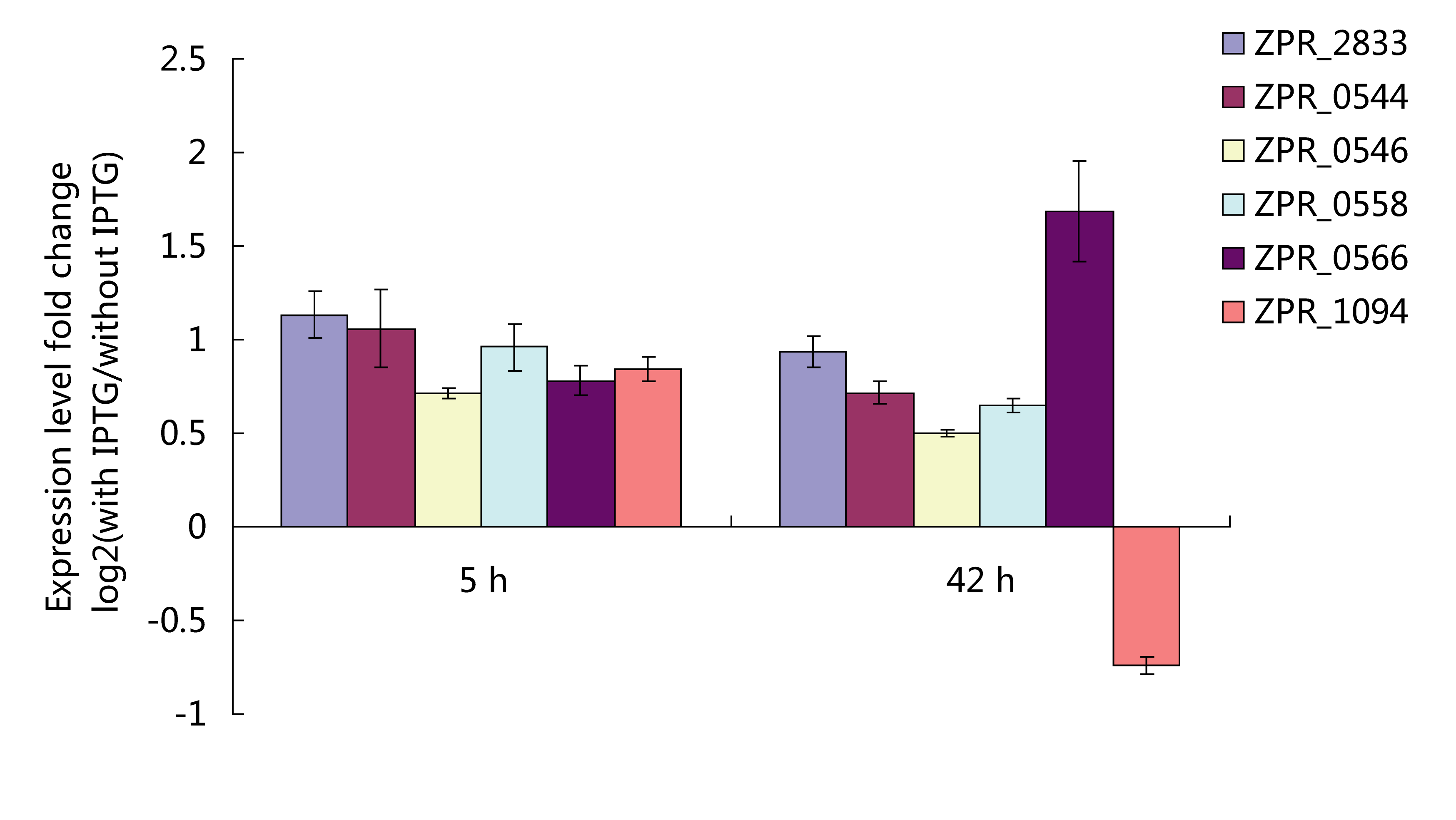


**Figure S5. The differential expression of target genes of SM-A87 induced with IPTG as determined by RT-qPCR.** Strains were grown in the basal medium supplemented with sucrose and galactose. After 5 and 42 hours of supplement of IPTG, the cells were collected and RT-qPCR experiments were performed.
