# Supplementary material for: Comparative Transcriptome Analysis Reveals That Lactose Acts as an Inducer and Provides Proper Carbon Sources for Enhancing Exopolysaccharide Yield in the Deep-Sea Bacterium Zunongwangia profunda SM-A87
Source: PLoS One. 2015 Feb 13;10(2):e0115998. doi: 10.1371/journal.pone.0115998 (PMC4332637; doi:10.1371/journal.pone.0115998)
Supplement: S1 Table — (DOC) [file pone.0115998.s006.doc]

Table S1 Expression level of genes within the LPS gene cluster^a^

| Locus tag | Annotation | Scale of expression level | |
| --- | --- | --- | --- |
|  |  | L4/O4 | L6/O6 |
| ZPR_1091 | polysaccharide export outer membrane protein | 1.4 | 0.6 |
| ZPR_1092 | Ptk-like tyrosine-protein kinase | 1.1 | 0.8 |
| ZPR_1093 | S23 ribosomal protein | - | 0.3 |
| ZPR_1094 | O-antigen export system permease protein | - | 0.6 |
| ZPR_1095 | ABC transporter, ATP-binding protein | 0.7 | - |
| ZPR_1096 | UDP-Glycosyltransferase/glycogen phosphorylase | 1.3 | 1.2 |
| ZPR_1097 | glycosyl transferase, family 2 | 0.7 | 0.7 |
| ZPR_1098 | glycosyl transferase family 2 | - | - |
| ZPR_1099 | glycosyl transferase family 2 | 0.7 | 0.8 |
| ZPR_1100 | glycosyltransferase | 0.8 | 1.2 |
| ZPR_1101 | putative UDP-N-acetylglucosamine 2-epimerase | 0.4 | - |
| ZPR_1102 | UDP-Glycosyltransferase/glycogen phosphorylase | 0.7 | 0.7 |
| ZPR_1103 | glycosyl transferase family 2 | - | - |
| ZPR_1104 | glycosyl transferase family 2 | 0.7 | - |
| ZPR_1105 | glycosyl transferase, group 1 | - | 1.5 |
| ZPR_1106 | glycosyl transferase | - | 1.6 |
| ZPR_1107 | exoV-like protein | 1.6 | - |
| ZPR_1108 | integrase | 0.3 | 0.1 |
| ZPR_1109 | IS3/IS911 family transposase | - | 0.1 |
| ZPR_1110 | glycosyl transferase, group 1 | - | 0.6 |
| ZPR_1111 | UDP-Glycosyltransferase/glycogen phosphorylase | 1.5 | 1.3 |
| ZPR_1112 | asparagine synthetase | - | 1.2 |
| ZPR_1113 | N-acylneuraminate cytidylyltransferase | - | 1.3 |
| ZPR_1114 | formyl transferase | - | 1.7 |
| ZPR_1115 | polysaccharide deacetylase | 2.0 | - |
| ZPR_1116 | UDP-N-acetylglucosamine 2-epimerase | 1.8 | 0.8 |
| ZPR_1117 | N-acetylneuraminate synthase | 2.2 | 0.8 |
| ZPR_1118 | glycosyl transferase family protein | - | - |
| ZPR_1119 | glycosyl transferase family protein | - | 1.6 |
| ZPR_1120 | serine acetyltransferase-like protein | - | - |
| ZPR_1121 | glycosyl transferase, group 1 | - | - |
| ZPR_1122 | putative glycosyl transferase | 1.5 | - |
| ZPR_1123 | O-Antigen Polymerase | 0.6 | 0.5 |
| ZPR_1124 | glycosyl transferases group 1 | 1.2 | - |
| ZPR_1125 | glycosyl transferase, group 1 | 2.0 | - |
| ZPR_1126 | sugar transferase | - | - |

^a^, ‘-’ denotes that the change of expression level is not significant.
